# Treatment of neuromyelitis optica/neuromyelitis optica spectrum disorders with methotrexate

**DOI:** 10.1186/1471-2377-14-51

**Published:** 2014-03-15

**Authors:** Ramnath Santosh Ramanathan, Konark Malhotra, Thomas Scott

**Affiliations:** 1 Department of Neurology, Drexel University College of Medicine, Allegheny General Hospital, 4742 Centre Avenue, Apt 703, Pittsburgh, PA 15213, USA

**Keywords:** Neuromyelitis optica, Neuromyelitis optica spectrum disorders, Methotrexate, Immunosuppressant therapy

## Abstract

**Background:**

To review our experience using methotrexate as a single long-term immunosuppressant (IS) therapy in neuromyelitis optica/neuromyelitis optica spectrum disorders (NMO/NMOSD).

**Methods:**

We performed a retrospective chart review of all patients with a diagnosis of NMO/NMOSD, supported by a positive NMO-IgG testing, who were treated with methotrexate. A paired sample 2 tailed t test was used to assess the annualized relapse rate during 18 months pre treatment with methotrexate to annualized relapse rate 18 months post treatment with methotrexate.

**Results:**

We followed 9 patients meeting criteria for the study for a median of 62 months. All patients were stabilized during attacks with high-dose steroids and/or plasmapheresis. Five patients (55.55%) were started on methotrexate as an initial long-term immunosuppressant strategy. Three patients (33.33%) were initially treated with pulse cyclophosphamide followed by methotrexate as a preplanned step-down strategy. One patient was started on azathioprine prior to methotrexate. No patient had side effects requiring change in methotrexate therapy. Five patients (55.55%) had stabilization of Expanded Disability Status Scale (EDSS) on methotrexate. One patient had a small increase in EDSS due to concomitant illness. Three patients (33.33%) had methotrexate treatment failure evidenced by worsening EDSS and ongoing relapses while on methotrexate, mandating a change in methotrexate therapy. Average annualized relapse rate in the entire group comparing 18 months prior versus 18 months after methotrexate treatment was reduced by an absolute value of 64% (3.11 vs 1.11). A paired t-test showed this reduction was highly significant (p = .009).

**Conclusion:**

In our experience, methotrexate is safe and possibly efficacious as a single long-term IS therapy along with low dose corticosteroids that can reasonably be offered to patients with NMO/NMOSD.

## Background

Neuromyelitis Optica (NMO) is a severe demyelinating inflammatory disease of the central nervous system characterized by recurrent attacks of myelitis and optic neuritis [1-3]. Evidence suggests aquaporin 4-antibodies (AQP4-ab) are primarily involved in the disease pathogenesis [1,4,5]. AQP4-IgG is predominantly IgG1 and is capable of activating complement, leading to blood brain barrier disruption and destruction of astrocytic membranes [6]. Recently it has also been shown that interleukin 6 (IL-6) plays a critical role in the pathogenesis of NMO [7,8].

It has been proposed that NMO/NMOSD requires long-term treatment with immunosuppressant therapy [9]. The largest case series suggest treatment of NMO/NMOSD with immunosuppressant (IS) therapy with rituximab, azathioprine and mycophenolate mofetile [10-12]. In these retrospective studies, 14 (56%) of 25 patients were relapse free with rituximab after a median follow up of 19 months, 37 (37%) of 99 patients were relapse free with azathioprine after a median follow up of 24 months, and 14 (58%) of 24 patients were relapse free with mycophenolate mofetile after a median follow up of 28 months [10-12]. A recent open label pilot study by Pittock et al. has shown the use of eculizumab, a humanized monoclonal IgG that neutralizes complement protein C5, had lead to relapse free state in 12 of the 14 patients over the period of 12 months [13]. The use of tocilizumab, a humanized monoclonal antibody directed against the IL-6 receptor, has been reported in a patient with severe NMO with improvement in EDSS scores [14].

Minimal data exists on methotrexate as a long-term treatment for NMO and NMOSD [15,16]. There are multiple proposed mechanisms of action of methotrexate to explain its efficacy in the attenuation of autoimmune illness, including inhibition of purine metabolism, interference with interleukin-1 beta binding to interleukin-1 receptors and interference with T-cell adhesion [17]. Considering patients with NMO/NMOSD will likely need many years of immune suppression, the long-term safety record of methotrexate makes this agent an attractive candidate for use in our clinic. We present our clinic experience of patients with NMO/NMOSD, treated with methotrexate, overviewing 9 patients.

## Methods

This retrospective analysis was approved by the Institutional Review Board of Allegheny General Hospital. We reviewed medical records of all patients with NMO (2006 diagnostic criteria) or NMOSD (patients who had one or more attacks of optic neuritis only, or transverse myelitis only and who were NMO-IgG seropositive) who were treated with methotrexate as IS therapy (2000 –2012). For each patient we recorded 1) demographics; 2) baseline clinical information; 3) treatment details (use of methotrexate during remission and relapse, timing of methotrexate initiation, concomitant corticosteroids, CBC, liver function tests, adverse effects, timing and reasons for discontinuation); 4) clinical course to last follow-up (dates of attacks and EDSS during remission and after relapses). A paired sample 2 tailed t test was used to assess the annualized relapse rate during 18 months pre treatment with methotrexate to annualized relapse rate 18 months post treatment with methotrexate.

## Results

Demographic information for the nine patients studied, EDSS scores and relapse summary information on the 9 patients are given in Table 1. Patients were followed for a median of 62 months (mean = 82.89, SD = 43.779) and were treated with methotrexate for a median of 29 months (mean = 40 months, SD = 20.005). 2 patients (22%) presented with optic neuritis as an initial attack, while 7 patients (78%) had myelitis initially, with 4 (57%) of the myelitis onset cases remaining classified as NMOSD throughout follow up (no optic neuritis). Patients were not retested for NMO IgG positivity following treatment.

**Table 1:** Demographic information for the 9 patients studied, EDSS scores and relapse summary information

**No.**	**Age**	**Gender**	**Months followed**	**1st attack**	**Type of relapses**	**Relapses before starting MTX**	**Relapse on MTX**	**Treatment history**	**Months on MTX**	**Initial EDSS**	**Final EDSS**
1	40	M	62	Myelitis	Myelitis	4	2	Stabilized on CTX* for 6 months, MTX** started as step down strategy, continued during last visit.	54	6.5	1.5
2	71	F	13	Optic Neuritis	Optic Neuritis and Myelitis	10	0	MTX started initially, continued during last visit.	11	5.5	3
3	62	F	146	Myelitis	Myelitis	5	1	MTX started initially, changed to MMF^^ briefly but due to S/E again started on MTX.	24	6.5	6.5
4	38	F	143	Myelitis	Optic Neuritis and Myelitis	7	0	Stabilized on CTX for 6 months, MTX started as step down strategy, continued during last visit.	60	4	4
5	46	F	114	Optic Neuritis	Optic Neuritis and Myelitis	8	2	Initially on AZA*** then following relapses MTX started, continued during last visit.	29	4	4
6	74	F	62	Myelitis	Myelitis	4	2	MTX started initially, continued during last visit.	52	4.5	5.5
7	54	F	85	Myelitis	Optic Neuritis and Myelitis	4	3	Stabilized on CTX for 6 months. MTX started as step down strategy, changed MTX to MMF due to increasing relapses and later to RTX^^^.	24	2	4.5
8	57	M	59	Myelitis	Myelitis	2	3	MTX started initially, MTX changed to MMF following continuing relapses and later to RTX.	7	3.5	5.5
9	52	F	62	Myelitis	Optic Neuritis and Myelitis	3	4	MTX started initially, RTX following continued relapses.	52	2.5	2.5

*CTX-cyclophosphamide, **MTX-methotrexate, S/E-side effect, ***AZA-azathioprine, ^^MMF-mycophenolate mofetile, ^^^RTX-rituximab.

**Figure 1:**
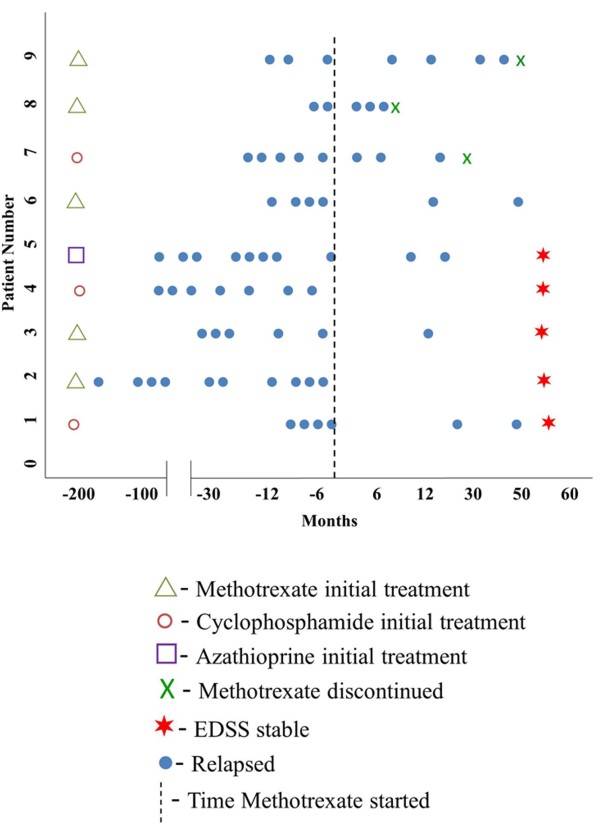
Depicting à summary of the treatment history, relapses and treatment outcome of all 9 patients in the study.

A summary of the treatment history of all 9 patients is shown in Figure 1. All patients received high-dose corticosteroids intravenously and/or plasmapheresis for initial attacks or relapses and were otherwise continued on low-dose corticosteroids (5–10 mg per day) along with methotrexate therapy throughout the treatment period. We treated patients with initial methotrexate dosage of 7.5 mg once weekly with titration up to a maximum of 17.5 mg once weekly, over several weeks. All 9 patients received pulse intravenous methylprednisolone 500 mg twice daily for relapses with oral conversion to 30 to 60 mg prednisone daily for 4–6 weeks followed by a steroid weaning protocol in which prednisone was tapered to 20–30 mg for 4–6 weeks followed by 10–20 mg for 4–6 weeks and all 9 patients were then maintained on low dose prednisone 5–10 mg for long term (at least 6 months).

Relapses were defined based on clinical deterioration of baseline symptoms or appearance of new symptoms with change in EDSS, after infection had been ruled out, hence all were true relapses. Five patients were initially started on therapy with methotrexate after stabilization with high-dose corticosteroids and/or plasmapheresis. Three patients were also initially treated with pulse cyclophosphamide (700 mg/M2) monthly for 6 months, with a planned step down therapy to methotrexate. One patient was started on azathioprine prior to methotrexate. Patients who were continued on methotrexate for their entire follow up (6/9, 67%) responded to treatment as evidenced by stability or improvement on EDSS, except 1 elderly patient had subtle and slow worsening of gait over 36 months to add a single-point to her EDSS score. Two of these patients had improvement of EDSS, and three had unchanged EDSS. The visual subscores on all the 9 patients were reviewed and we found that of 5 patients with optic neuritis and myelitis, 2 patients had visual FSS (functional system scores) of 5 pre and post methotrexate treatment (both patients had unilateral blindness, with visual acuity of 20/20 in the other eye), 2 patients had visual FSS of 0, and 1 patient had visual FSS of 1 with no change in visual subscores post treatment with methotrexate.

Two of the patients who were continued on methotrexate had zero relapses, while three had 2 relapses each, with these relapses being easily managed with full recovery. Three patients (33.33%) were considered to be treatment failures during treatment with methotrexate due to multiple relapses (greater than or equal to three each) and methotrexate was changed to rituximab resulting in stabilization. None of the patients developed any signs or symptoms of toxicity while on methotrexate with complete blood count and liver function tests being monitored every 8–12 weeks. Average annualized relapse rate in the entire group comparing 18 months prior versus 18 months after methotrexate treatment was reduced by an absolute value of 64% (3.11 vs 1.11). A paired t-test showed this reduction was highly significant (p = .009).

## Discussion

This cohort represents the 3^rd^ series of patients with NMO/NMOSD studied retrospectively after treatment with methotrexate and is comparable with data published by Kitley et al. [15,16]. Statements concerning efficacy in this scenario of a retrospective cohort are scientifically weak, but are reasonably afforded some validity given the lack of superior studies. The lack of relapses or relapses with residual effects seen in several of our patients is somewhat encouraging in this regard, providing efficacy data on par with some previous case series. We acknowledge significant selection bias as well as the limitations of EDSS as an assessment tool in NMO.

Individual cases in our series can give clinicians insight into patterns of relapse and progression seen in NMO. For example, in patient 1 EDSS reached to 6.5 due to relapses prior to starting MTX, at which point the patient received treatment with high dose IV corticosteroids and monthly pulse cyclophosphamide for 6 months with planned step down strategy to methotrexate and maintenance low dose steroids. Improvement was seen steadily over several months with documented improvement of EDSS to 1.5. EDSS improvement over many months implies good response to cyclophosphamide, followed by methotrexate and steroids over a protracted period. Similarly patient 2 had 10 relapses prior to starting methotrexate but had a sustained relapse free period with improvement in EDSS following treatment with methotrexate and low dose maintenance steroids. Furthermore, overviewing our entire case series, although complete freedom from relapse is a goal, it is not clear whether or not relapse free status is needed to achieve progression free status (some patients were not relapse free but quickly responded to a single high dose corticosteroid pulse to return to baseline).

In patients with severe onset of NMO/NMOSD we have tended to use therapeutic agents which have already had reported success in reducing relapses (rather than methotrexate which has been minimally used in this disorder). We suspect that such patients are more vulnerable to further severe attacks. Contra wise, in patients with mild onset, given the perceived need for long-term immunosuppression in patients with NMO/NMOSD, and given the excellent safety profile of methotrexate, we feel it is reasonable to attempt treatment with methotrexate as initial therapy. We also used methotrexate as a “step down” therapy from other treatments, which are possibly more toxic (cyclophosphamide in 3 cases). Elderly patients, being perhaps more susceptible to side effects of chronic immune suppression, may also be good candidates for initial therapy with methotrexate. Reviewing the long-term safety data on the handful of immunosuppressant agents commonly used in NMO/NMOSD, we find methotrexate has an excellent safety profile. Safety data for long-term use of mycophenolate mofetile, azathioprine, and rituximab might be interpreted as inferior or less robust by comparison. Extensive experience with rheumatoid arthritis and other autoimmune illness has provided an extensive amount of safety data on methotrexate [18].

Available data from other cohort studies suggest rituximab may be a particularly effective first-or second-line agent for NMO/NMOSD. We lean towards the use of rituximab for treatment failure after initial attempts with agents such as mycophenolate mofetile, azathioprine, methotrexate and cyclophosphamide, as evidenced in our present series. It is of interest that in our patients who failed therapies before rituximab, stabilization ensued after changing to this monoclonal antibody. Head to head trials of rituximab versus other promising agents (like azathioprine, mycophenolate mofetile or methotrexate) for treatment of NMO/NMSD are deemed to be difficult to perform due to the rarity of the disorders, the long term follow up that is needed and the severe consequences of treatment failure among other factors. However, controlled trials are needed.

## Conclusion

Our experience has shown that methotrexate is a safe single IS therapy along with low dose corticosteroids, which can possibly be used efficaciously in patients with NMO/NMOSD for long-term management.

## Competing interests

The authors declare that they have no competing interests.

## Authors’ contributions

RSR participated in the data collection, design and interpretation of the study as well as preparation of the manuscript. KM participated in the design, interpretation of the study and preparation of the manuscript. TS participated in data collection, design and interpretation of the study as well as preparation of the manuscript. All authors read and approved the final manuscript.

## Pre-publication history

The pre-publication history for this paper can be accessed here:

http://www.biomedcentral.com/1471-2377/14/51/prepub
